# Editorial: Psychosocial Job Dimensions and Distress/Well-Being: Issues and Challenges in Occupational Health Psychology

**DOI:** 10.3389/fpsyg.2017.02213

**Published:** 2017-12-18

**Authors:** Renato Pisanti, Anthony J. Montgomery, James Campbell Quick

**Affiliations:** ^1^Department of Psychology, University Niccoló Cusano, Rome, Italy; ^2^Department of Education and Social Policy, University of Macedonia, Thessaloniki, Greece; ^3^Goolsby Leadership Academy, University of Texas at Arlington, Arlington, TX, United States; ^4^Organizational Psychology, Alliance Manchester Business School, The University of Manchester, Manchester, United Kingdom

**Keywords:** psychosocial job stress, occupational distress, occupational health, employee health, job stress models

Over the past 40 years, few topics in the organizational psychology have attracted as much attention as the impact of psychosocial job dimensions on psychological distress and well-being. Theoretical models, such as the effort-reward imbalance (ERI) model and the job demands-resources (JD-R) model suggest that distress and positive dimensions at work (well-being and motivation) can be considered as two sides of the same coin. If the job is designed to provide the right mix of psychosocial job dimensions (e.g., optimal time pressure and decision authority), work can boost job engagement and well-being as well as productive behaviors at work. When the job is not designed in an optimal way (e.g., too much time pressure and too little decision authority) work can trigger stress reactions and burnout.

The aim of this research topic was to bring together international research from different theoretical and methodological perspectives in order to advance knowledge and practice in the field of occupational stress. This e-book presents twenty papers that cover a range of topics, from burnout to illegimate tasks, and a range of methodologies from diary studies to meta-analysis. The papers provide new insights into the JD-R and ERI models, the Conservation of Resources theory, recovery from work, detachment and job satisfaction.

The e-book starts with a review paper written by Zacher and Schmitt, in which the authors review research on the role of age in the relationship between psychosocial job dimensions and occupational well-being. Their review considers the following theoretical frameworks: the lifespan perspective on work design, person-environment interaction and fit theories, as well as models of successful aging at work, to explain the interaction effects of psychosocial job dimensions and age on occupational well-being. Furthermore, role theory has been adopted primarily to clarify why psychosocial job dimensions may mediate associations between age and occupational well-being indicators. The authors conclude that relationship of age with specific work characteristics and occupational well-being indicators can be linear or non-linear. A series of original studies present promising extensions to the JD-R model (Bakker and Demerouti, 2017). The JDR model assumes that job demands and resources trigger two processes; namely a health-impairment process and a motivational process. In the first paper Molino et al. examine, among call center employees, the mediational role of emotional dissonance between job demands (workload and customer verbal aggression) and job resources (supervisor support, colleague support, and job autonomy) on the one hand, and, on the other, affective discomfort. Emotional dissonance derives when employees should express emotions considered acceptable by the organization, but do not represent the true feelings of the individual. Their results illustrate that only for the subgroup of agents who had to provide technical and specific customer service, emotional dissonance fully mediated the relationship between workload and affective discomfort, and it partially mediated the relationships between customer verbal aggression and job autonomy on the one hand, and affective discomfort on the other. Ceschi et al. examine whether job demands and job resources can moderate the relationships between two decision making dimensions (decision making competency and decision environment management) and job performance (in-role and extra-role performance). Results show that among employees who perceive low levels of job demands, decision making competency is positively related to in-role performance; whereas such an interaction disappears for high levels of job demands and decision making competency. Furthermore, employees who perceive high levels of decision environment management and high levels of job resources are more prone to perceive high levels of extra-role performance than their counterparts who perceive high (or) low decision environment management and low job resources. Baeriswyl et al. examines the role of work family conflict as an intervening variable in the JD-R model among airport security officers employed at a European airport. Work family conflict occurs when job demands interfere with family domain such as irregular work hours, work overload, etc. Work family conflict partially mediated the impact of supervisor support and workload on job satisfaction and emotional exhaustion. Finally, two longitudinal studies examine both the direct and moderating effects between job demands and resources variables on burnout dimensions (emotional exhaustion, depersonalization/cynicism, and personal accomplishment). Pisanti et al. found, in a sample of nurses, that unfavorable changes in psychosocial job dimensions are associated with increases of burnout variables over time. Also, Jimenez and Dunkl, in a sample of workers employed in different industrial sectors (e.g., manufacturing), found cross lagged effects of job characteristics on burnout dimensions. In both studies the moderating effects of job resources were found for the dimension personal accomplishment. All five papers further extend the theoretical and practical knowledge base concerning the JD-R model.

Three further articles focus on the Conservation Of Resources (COR) theory (Hobfòll, 2001). The main assumption of the COR theory is that a loss or the potential loss of resources are psychologically threatening. In the Lee et al. paper, the direct associations of negative and positive work-to-family spillover on emotional exhaustion and job satisfaction are analyzed at the individual and organizational level in a group of hotel managers', beyond the effects of job demands and supervisors' leadership style. Moreover, the authors examine the cross-level (individual vs. organizational) interactive effect of negative work-to-family spillover and positive work-to-family spillover. The authors found that beyond the effects of psychological job demands and supervisor's transformational leadership, at the individual level, hotel managers who perceive higher negative work-to-family spillover report more exhaustion and lower job satisfaction, whereas those with higher positive work-to-family spillover perceive less exhaustion and higher satisfaction. The negative link between individual-level negative work-to-family spillover and job satisfaction is moderated when organization-level positive work-to-family spillover is higher, compared to when it is lower. The issue of work and family, as interconnected spheres of life that play a vital role in employee well-being, is also examined in the paper of Dåderman and Basinska. In a group of female nurses, the authors examine direct effects between job demands and engagement on the one hand, and, on the other, turnover intentions. Furthermore, the authors analyze whether levels of work family conflict and family work conflict moderate the associations between job demands and engagement with turnover intention. They found that only high job demands and low vigor were significantly associated with turnover intentions. Rogala et al. in two longitudinal studies, highlight the role of self-efficacy beliefs as personal resources that mediate the association between emotional exhaustion and disengagement. In both studies these associations were mediated by self-efficacy: higher exhaustion may trigger a spiral loss of self-efficacy which in turn may lead to higher disengagement at follow-up.

A series of studies on the role of detachment and recovery come next. The first is a meta-analysis by Wendsche and Lohmann-Haislah, in which the authors examine the antecedents and outcomes of detachment from work. The meta-analysis indicated that average relationships between detachment and physiological stress indicators and work motivation were not significant while associations with contextual performance and creativity were significant, but negative. Moreover, results indicated that psychological detachment was negatively related with individual dimensions such as negative affectivity and heavy work investment. Germeys and De Gieter present the results of a daily diary study conducted among 136 employees during 10 consecutive working days. The main focus of their research concerns the positive effects on the well-being of the daily psychological detachment from work. Psychological detachment fully mediated the daily relationship between workload and marital satisfaction. In the paper of Cropley et al. the authors present three original studies focused on the relationships between work-related rumination and cognitive processes (such as planning and working memory) centered on the construct of executive functioning. Previous studies (i.e., Cropley and Zijlstra, 2011) have defined rumination as the process of perseverative thinking or dwelling about problems and issues relating to work. Overall, the three studies demonstrate significant associations between work-related rumination and measures of executive functioning such as cognitive failures, cognitive flexibility, and situational awareness at work. Moreover, “High ruminators” showed greater difficulties with “lapses of attention,” “lack of focus of attention,” and “absent mindedness”; rather than “Low ruminators.” Finally, high work-related rumination was associated with other central dimensions of executive functioning such as deficits in starting and finishing projects, memory, and feeling compelled to do things. The authors conclude their paper by arguing that work-related rumination may not be related to work demands *per se*, but appears to be an executive functioning/control issue. Overall, the papers provide interesting insights regarding recovery strategies and psychological detachment from work.

Two further papers focus on illegitimate tasks. Tasks may be viewed as illegitimate to the extent that they are perceived to be unnecessary or unreasonable. They imply a threat to one's professional identity. These tasks arouse feelings of injustice because they are perceived as infringing the norms about what can be expected from an employee (Semmer et al., 2015). Faupel et al. present the results of a study conducted on the basis of qualitative content analysis. They present typical illegitimate tasks in the context of teacher training. Unnecessary tasks could be categorized as sub-challenging (e.g., “We all studied and learned these things at university.”), inefficient and lacking in organization. Unreasonable tasks relate to ones that overextend us, fall outside our area of responsibility, and lack appropriate supervisory support. Omansky et al. among a sample of 213 employees in various predominantly junior-level positions, demonstrate that illegitimate tasks are significantly and negatively associated with job satisfaction and intrinsic motivation. Moreover, a moderated-mediation effect was found such that male workers reacted more than female workers to illegitimate tasks through the mechanism of perceived ERI. Overall, these two papers highlight the importance of including illegitimate tasks as new forms of occupational stressors in future studies.

Schulz et al. provide an original perspective by examining a multilevel model where team health climate is hypothesized to be associated with health-related outcomes (i.e., subjective general health, psychosomatic complaints, mental health, and presenteeism). Team health climate is defined as “employees' shared perceptions of the extent to which their team is concerned, cares, and communicates about health issues” (Schulz et al.). Team health climate was associated with all health outcomes (except psychosomatic complaints), above and beyond the effects of other dimensions (e.g., team size, job demands, job control, and employees' individual perceptions of health climate). Furthermore, analyses reveal that a positive team health climate may buffer the negative relationship between employee age and work ability. The authors describe interesting implications for occupational health interventions in teams.

San-Martín et al. in a sample of healthcare workers, describe the relationships between professionalism (a comprehensive construct composed of empathy, teamwork, and lifelong learning) and distress dimensions (somatization, exhaustion, and work alienation). The authors describe important differences in the pattern of correlations between subgroups of physicians and nurses. Furthermore, the research reveals interesting findings concerning gender (higher somatization in female physicians and nurses than in male groups), and professional status (higher exhaustion and alienation in physicians than in nurses).

Two papers focus their attention on a central construct in occupational health psychology: job satisfaction. In the first paper Sola-Carmona et al. describe the associations between job satisfaction, family satisfaction, and material well-being in a group of parents with blind children. Results highlight a positive correlation between job satisfaction and material well-being, and between material well-being and family satisfaction. However, no statistically significant correlation is found between job satisfaction and family satisfaction. The authors argue that it is necessary to provide families with more economic resources, which would have a positive impact on their subjective psychological well-being, decreasing their state-anxiety, and increasing their satisfaction with life. In the second paper; Unanue et al. present the results of three studies which describe the association between job satisfaction and life satisfaction. A first series of results regards the positive association between job satisfaction and life satisfaction both contemporaneously and longitudinally, and vice-versa, above and beyond several key control demographic variables. Furthermore, the cross-sectional and longitudinal links between job satisfaction and life satisfaction are spurious, and instead they are rooted in basic psychological needs (as stated by self-determination theory) such as: need for satisfaction at work and need for satisfaction in general life.

Two final papers are focused on interventions. Keeman et al. combine the results from two studies carried out to evaluate a free online game aimed at encouraging wellbeing enhancing activities (Wellbeing Game). The game design is informed by the Five Ways to Wellbeing framework. The results showed that after playing the Wellbeing Game, employees reported lower stress levels, and higher well-being levels for those who felt that it had helped them connect more with colleagues. The authors conclude that the studies present preliminary evidence for this type of positive gain spiral but more longitudinal data are needed to examine this further. Finally, Abildgaard et al. focus on the methods to improve the assessment of the intervention process. They use the quantitative and qualitative date from an organizational intervention conducted in a national postal service. The authors describe what information about the intervention process is to be gained from quantitative and qualitative process evaluation and examine strengths and weaknesses as well as potentials for mixed methods evaluation methodologies.

The 20 papers, included in the present e-book, provide new avenues for future research and further refine our understanding of the theoretical and practical issues involved in understanding how psychosocial job dimensions and well-being are linked.

## Author contributions

All authors listed have made a substantial, direct and intellectual contribution to the work, and approved it for publication.

### Conflict of interest statement

The authors declare that the research was conducted in the absence of any commercial or financial relationships that could be construed as a potential conflict of interest.
